# Utilization of information and communication technology (ICT) among undergraduate health science students: a cross-sectional study

**DOI:** 10.1186/s12909-022-03296-9

**Published:** 2022-03-30

**Authors:** Samuel Hailegebreal, Tigists Tolessa Sedi, Selamawit Belete, Kirubel Mengistu, Anteneh Getachew, Daniel Bedada, Mebrie Molla, Tamiru Shibiru, Shegaw Anagaw Mengiste

**Affiliations:** 1 Department of Health Informatics, School of Public Health, College of Medicine and Health Sciences, Arba Minch University, Arba Minch, Ethiopia; 2 School of Medicine, College of Medicine and Health Sciences, Arba Minch University, Arba Minch, Ethiopia; 3 University of South-Eastern Norway, Post office box 235, N-3603 Kongsberg, Norway

**Keywords:** Utilization, Information, Communication, Technology, Health science student

## Abstract

**Background:**

We already know that incorporating information and Communication technology (ICT) into every aspect of human activity result in significant change and makes tasks easier to complete. It can help in areas of healthcare systems and medical education. Therefore, this study aimed to assess utilization ICT and its associated factors among Arba Minch University College Medicine and Health Science students.

**Methods:**

A cross sectional study design was conducted in June through August 2021 among under graduate students in college of medicine and health science at Arba Minch University, Ethiopia. A self-administered questionnaire was used to collect information on the students’ socio-demographic factors as well as the utilization ICT. The data entry form was prepared with Epi-data 3.1 versions software and STATA version 14 software was used to analyze the data.

**Results:**

A total of 355 participants enrolled in the study, with a response rate of 98.34%. The percentage of students who used ICT was 55.77% [95% CI, 0.50, 0.60]. Regarding of field of study, health informatics students (84%) used the most ICT, while midwifery students (52%) used the least. Urban resident [AOR = 1.85, 95% CI = 1.08, 3.16], ICT knowledge [AOR = 3.8, 95% CI = 2.25, 6.40], having formal training of ICT [AOR = 1.9, 95% CI = 1.06,3.48], having IT in current course study [AOR = 2.2, 95% CI = 1.23, 3.84], and had good IT skill [AOR = 2.4, 95% CI = 1.34, 4.23] revealed a significant and positive correlation with the use of ICT.

**Conclusion:**

In the current study previous residence, ICT knowledge, having formal training, having IT in current courses, and IT skill were significantly associated with student ICT utilization. Therefore, the university should continue to invest in professional development in order to improve teaching and student performance, as well as provide the college with student-centered ICT computer labs to encourage students to use technology.

## Introduction

In today’s society, information and communication technologies (ICT) play an important part in almost every area [1, 2]. ICTs have the potential to promote patient-centered healthcare at a reduced cost, increase quality care and information sharing, educate health professionals and patients, stimulate a different sort of interaction with patients and health providers, and minimize travel time [3, 4]. The healthcare system is growing increasingly dependent upon technology, so, health science students are expected to improve their skills of ICT [5, 6]. Also, ICT enables the use of novel educational materials and the renewal of learning methods, allowing students to collaborate more actively and simultaneously acquire technological expertise [7]. In spite of the fact that there’s no single, widespread definition of ICT, the term is for the most part acknowledged to cruel all devices, organizing components, applications and frameworks that combined permit individuals and organizations associated within the computerized world [8].

While we understand that the use of educational technologies in higher education teaching and learning activities is still in its infancy in Ethiopia, ICT instructional use is critical to both faculty and student success and development [9]. In education, the use of ICT to improve or support learning and teaching has grown increasingly significant [10, 11]. For several decades, many authors have argued that ICT as educational devices facilitate the adaptation of teaching to each student [9, 12]. Although it is widely assumed that ICT can empower teachers and students, encourage change, and nurture the development of ‘twenty-first century skills, data to back up these claims is still scarce ( [9], Https://www.infodev.org/innovationandEntrepreneurship). ICT in health care, and both educational opportunities and assistance for students and health care professionals have expanded [13, 14]. The COVID-19 epidemic has had a significant impact on all aspects of schooling, particularly in light of existing social distancing norms. Medical educators are employing a variety of information and communication technology (ICT) methods to maintain medical education in the face of the pandemic. The majority of medical educators use university websites and online collaboration technologies to disseminate study materials [15, 16].

Computer technology and digital world have revolutionized the way people live, work, development and distribution of knowledge and power around the world (Https://learningportal.iiep.unesco.org/en.learning portal,2021). Because the integration of informatics into health science education is becoming necessary in many universities across the world, it is critical to assess health science students’ factors to use computers and ICT technologies [17–19]. Expanded usage of eHealth is projected in Ethiopia in the future years, appreciations to new efforts, but these systems must be used properly to accomplish objectives; this is entirely dependent on health science student since they will be professional in few years later [20]. Therefore, the objective this study was to assess utilization of information and Communication technology and its associated factors among undergraduate health science student.

## Methods

### Study design, setting, and period

A cross sectional study design was conducted in June through August 2021 among under graduate students in college of medicine and health science at Arba Minch University, Ethiopia. Arba Minch University College of Medicine and Health Sciences, was founded in 2008. It is located in Ethiopia, SNNP regional State, Gamo Gofa zone, Arba Minch town is located around 434.3 kms from Addis Ababa, the capital city of Ethiopia. It has been focusing on its fundamental mission of operating programs that earnestly complement and augment the nation’s healthcare needs since its foundation. Under college of medicine and health science there are 10 departments running undergraduate study programs.

### Study population

This study included all regular undergraduate students from Arba Minch University College of Medicine and Health Sciences who were available during the data collection period and were in their second year or above were included in this study.

### Sample size determination and sampling procedure

The sample size was calculated using single population proportion formula with the following assumptions: 95% confidence level (CI), Z (1-α/2) =1.96), based on previous studies proportion of ICT utilization (*p* = 30.9%) [21], and 5% margin of error by considering a 10% non-response rate. Finally, 361 full-time undergraduate students were enrolled in this study. In Arba Minch University, college of medicine and health science, there are 10 departments (i.e. medicine, public health officer, medical laboratory sciences, midwifery, health informatics, anesthesia, radiology, pharmacy, environmental health and nursing). Later on, the proportional allocation was used for each department and academic year. Finally, using a student attendance list, a simple random sampling technique was used to withdraw study participants.

### Data collection

Structured self-administered questionnaires were adapted after reviewing relevant literature [21–23]. Pretest for the questionnaire was made on about 5% of the total studied population at Wolaita Sodo University which is outside of the study area and used to collect data from the study participants. The questionnaire comprised of socio demographic and basic IT skills and knowledge of computer hardware, software, computer input devices, computer output devices, basic computer words and definitions, and a general concept of how a computer works were assessed by a questionnaire. A total of 16 questions on ICT knowledge were asked, with each statement having a yes or no question to assess the level of ICT knowledge. In this study, participants were also asked about ICT Utilization questions to determine their level of ICT usage. In this study, respondents who scored mean value and above for utilization related questions were rated as good utilization rate; those who scored below mean value for a set utilization related questions were rated poor utilization rate.

### Study variables

The dependent variable for this study was utilization of ICT.

Independent variables sex, age, year of study, previous residence, field of study, parental educational status, family monthly income, previous IT training status, personal computer ownership, current information technology course taken, IT knowledge, availability of computer lab session, IT skill.

### Statistical analysis

Epi-data 3.1 versions used for data entry and STATA version 14software was used to analyze the data. Descriptive statistics was computed to describe socio demographic characteristics. Bivariate and multivariable logistic regression analysis was done to identify factors associated with ICT utilization. To control the possible effect of confounders, variables from the bivariate logistic regression with a *p*-value less than 0.25 were fitted into the multivariable logistic regression. Model fitness was checked by the Hosmer-Lemeshow goodness of-fit test. Finally, the results were interpreted using an adjusted odds ratio (AOR) with a 95% confidence interval (CI).

### Ethical issues and approval

Ethical clearance obtained from Arba Minch University, College of Medicine and Health Sciences, Institutional Research Ethics Review Board (IRB). Written and signed voluntary informed consent obtained from all study participants. All methods were performed in accordance with the relevant guidelines and regulations.

## Result

### Socio-demographic characteristics of study population

A total of 355 participants enrolled in this study, with a response rate of 98.34%. Majority 239 (67.3%) of respondents were from urban residents. More than half of the respondents (54.08%) were men. The majority of the participants (75.8%) were between ages group of 21–24. Majority of the participant were from medicine (35.2%) department followed by nursing (10.4%) and public health (10.4%). Regarding to family educational status 61.4% fathers and 50.7% mothers had secondary and above educational levels (Table 1).

**Table 1:** Demographic characteristics of the study population (*N* = 355)

Variable	Category	Frequency	Precent (%)
**Age**	15–20	50	14.08
	21–24	269	75.77
	25–30	36	10.14
**Sex**	Male	192	54.08
	female	163	45.92
**Previous residence**	Urban	232	65.35
	Rural	123	34.65
**Year of study**	Second	102	28.73
	Third	96	27.04
	Fourth	102	28.73
	Fifth	25	7.04
	Sixth	30	8.45
**Field of study**	Nursing	37	10.42
	Pharmacy	26	7.32
	Health informatics	19	5.35
	Public health	37	10.42
	Environmental health	19	5.35
	Anesthesia	16	4.51
	Radiology	15	4.23
	Medicine	125	35.21
	Medical laboratory	28	7.89
	Midwifery	33	9.30
**Father educational status**	Illiterate	30	8.45
	Read and write	56	15.77
	Primary	51	14.37
	Secondar and above	218	61.41
**Mothers’ educational status**	Illiterate	64	18.03
	Read and write	59	16.62
	Primary	52	14.65
	Secondar and above	180	50.70

### Access to computer and internet

In this study finding (23%) student had formal IT training access. From the total of study participant 63.9% stated that they have access to a computer, of this 50.7% had laptop, 9.6% palmtop, and 3.7% PAD respectively. From the total of study participant 87.6% had internet access, of this 31.3% the participant was access from computer laboratory and 10.7% from internet cafe. It was observed from the study that only 36.3% of the participant had laboratory session during their ICT course (Table 2).

**Table 2:** Access to computer and internet

Variable	Category	Frequency	Precent (%)
**Access formal IT training**	Yes	111	31.27
	No	244	68.73
**having personal computer**	Yes	227	63.94
	No	128	36.06
**Internet access**	Yes	311	87.61
	No	44	12.39
**IT course in current study**	Yes	175	49.30
	No	64	18.03
**Having lab session in IT course**	Yes	129	36.34
	No	226	63.66
**Access to electronic document in college**	Yes	282	79.44
	No	73	20.56

### Student ICT utilization

**Fig. 1:**
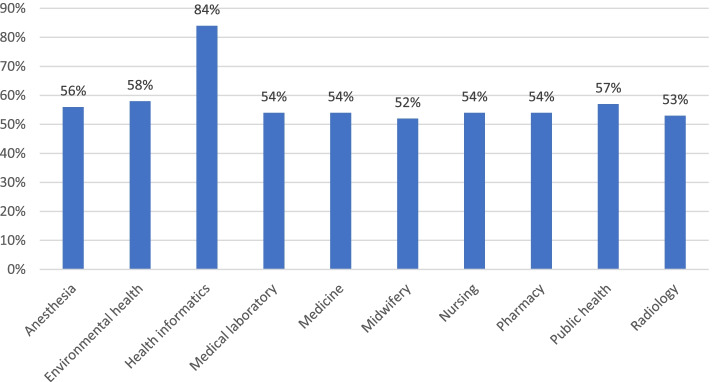
utilization of ICT based on field of study

In this study percentage of students who used ICT was 55.77% [95% CI, 0.50, 0.60]. In terms of field of study, health informatics students used the most, while midwifery students used the least (Fig. 1).

### Factors associated with ICT utilization

In the multivariable logistic regression model five variables; previous residence, ICT knowledge, having training, current IT courses taking, and IT skill were significantly associated with ICT utilization.

The odds of utilizing ICT among student who come from urban resident were 1.85 times [AOR = 1.85, 95% CI = 1.08, 3.16] higher than that of those come from rural resident. Students who had ICT knowledge were 3.8 times [AOR = 3.8, 95% CI = 2.25, 6.40] more likely to utilize ICT compared to their counterpart. Students who had formal training of IT were 1.9 times [AOR = 1.9, 95% CI = 1.06, 3.48] more likely to utilize ICT compared to those who had not formal IT training. Students who taking IT course in their present study were 2.2 times more likely to use ICT than students who did not take an IT course in their current study [AOR = 2.2, 95% CI = 1.23, 3.84]. Students who had good IT skill were 2.4 times more likely utilize ICT than those with poor IT skill [AOR = 2.4, 95% CI = 1.34, 4.23] (Table 3).

**Table 3:** Factor associated with the utilization of ICT among undergraduate students

Variable	Utilization		COR (95% CI)	AOR (95% CI)
	Yes	No		
**Age**				
15–20	26	50	1	1
21–24	107	162	1.64 [0.89,3.00]	1.75 [0.82, 3.71]
25–30	24	12	0.54 [0.22,1.31]	0.57 [0.19, 1.65]
**Previous residence**				
Rural	78	45	1	1
Urban	79	153	3.35 [2.12,5.29] **	1.85 [1.08, 3.17] *
**IT skill**				
have no skill	63	35	1	1
have skill	93	163	3.15 [1.94,5.12] **	2.38 [1.34, 4.23] **
**IT knowledge**				
Poor	90	41	1	1
Good	67	157	5.14 [3.22,8.20] **	3.80 [2.25, 6.40] **
**Formal IT training**				
No	131	113	1	1
yes	26	85	3.78 [2.28,6.28] **	1.93 [1.07, 3.48] *
**having personal computer**				
Yes	86	141	1	1
No	71	57	0.48 [0.31,0.76] **	0.69 [0.40, 1.21]
**IT course in current study**				
No	103	77		1
Yes	54	121	2.99 [1.93,4.63] **	2.18 [1.23, 3.84] **
**Having lab session in IT course**				
Yes	49	80	1	1
No	108	118	0.66 [0.43,1.04]	1.67 [0.91,3.07]

Key: 1: reference group; []: confidence interval, *p*-value 0.05–0.01 *: *p*-value < 0.01 **

## Discussion

The objective of this study was to examine utilization of information communication technology and its associated factors among Arba Minch University College of medicine and health science students. This study revealed that ICT utilization among student was 55.77% [95% CI, 0.50, 0.60], and 44.23% [95% CI: 0.39, 0.49] students didn’t utilize the ICT. This finding was greater than study from Gondar [22]. The temporal gap between globalization and technical improvement in recent years could be the reason. This finding was lower than study done in Jordan [24], Egypt [25] and Ghana [26]. This could be owing to limited access to IT resources in college, or because students were dissatisfied with computer laboratory sessions in terms of the number of computers accessible, the amount of time allowed for exercise and assistance from the laboratory assistant.

Besides, the inadequacy of computer laboratories and computers in the college, where there are only two computer labs, each with about 40 computers, as well as the majority of computers in the university were not working for all of the students, could explain the poor use of ICT.

In multivariable logistic regression analysis; previous residence, ICT knowledge, having formal training, taking IT in current courses, and IT skill were all found to be significantly associated with ICT utilization. When compared to students from rural areas, students from urban areas were more likely to utilize ICT. This finding was similar with previous study [27]. This might be due to that those students who come from rural resident don’t have access to electronic materials, because luck of infrastructure like electricity and computers. Students who had good ICT knowledge were more likely to utilize ICT as compared to poor ICT knowledge. This finding supported study done in Ghana [26]. The possible explanation could be it is therefore essential that students acquire ICT knowledge in order to ensure that ICT tools are used and adopted in order to facilitate efficient learning and teaching.

Regarding to students who had IT skills were more likely to utilize ICT than who had not skill. The finding was supported by similar study [11, 12]. This could be attributed to the fact that knowing how to use a range of computer programs, software, and other applications is an important IT ability. Word processing, spreadsheets, databases, PowerPoint presentations, and search engines are just a few examples of ICT applications that having IT skills may aid with. In this finding students who have taken IT course were more likely utilize ICT than their counterpart. This finding was supported by study from previous study [7]. This might be due to that taking IT related course may help to increase the use of computer and electronics materials. Also, formal information technology training was found to be significantly associated with ICT utilization [22, 28].

Furthermore, his finding revealed that student utilization of ICT was inadequate. Emphasis should be placed on helpful training in ICT as well as ICT-enabled teaching and learning. ICT should be skilled as a subject, and combined as a pedagogical tool for teaching and learning in other theme areas. Arba Minch University’s College of Medicine and Health Sciences might accordingly take advantage of respondents’ interest to learn more about ICT applications and create electronics health related courses for each of the schools.

### Limitations of the study

This study did not address the attitude of student towards ICT, which can influence their computer knowledge and utilization. Moreover, the information collected was self-perceived, which might have reported bias. The cross-sectional nature of study design may affect causality being inferred between independent and dependent variables.

## Conclusion

In the current study previous residence, ICT knowledge, having formal training, taking IT in current courses and IT skill were significant predictors of ICT utilization. The findings suggest that positive actions should be done to raise the level of ICT utilization among undergraduate students, such as the formal inclusion of ICT training in undergraduate student education. This will improve health science students’ ability to obtain, analyze, and use information in order to solve clinical and other problems promptly and efficiently throughout their studies and, more crucially, after graduation.

## Data Availability

The data in which the authors used to produce this manuscript are available upon reasonable request from the correspondence author.

## Abbreviations

AOR: Adjusted Odds Ratio
CI: Confidence Interval
ICT: Information Communication Technology
IT: Information Technology
OR: Odds Ratio
